# Supplementary material for: CXCR1- or CXCR2-modified CAR T cells co-opt IL-8 for maximal antitumor efficacy in solid tumors
Source: Nat Commun. 2019 Sep 5;10:4016. doi: 10.1038/s41467-019-11869-4 (PMC6728370; doi:10.1038/s41467-019-11869-4)
Supplement: Supplementary file 1 — Supplementary Information [file 41467_2019_11869_MOESM1_ESM.pdf]

Supplementary Information

CXCR1- or CXCR2-Modified CAR T cells Co-opt IL-8 for Maximal Antitumor Efficacy in Solid Tumors

Linchun Jin, et al

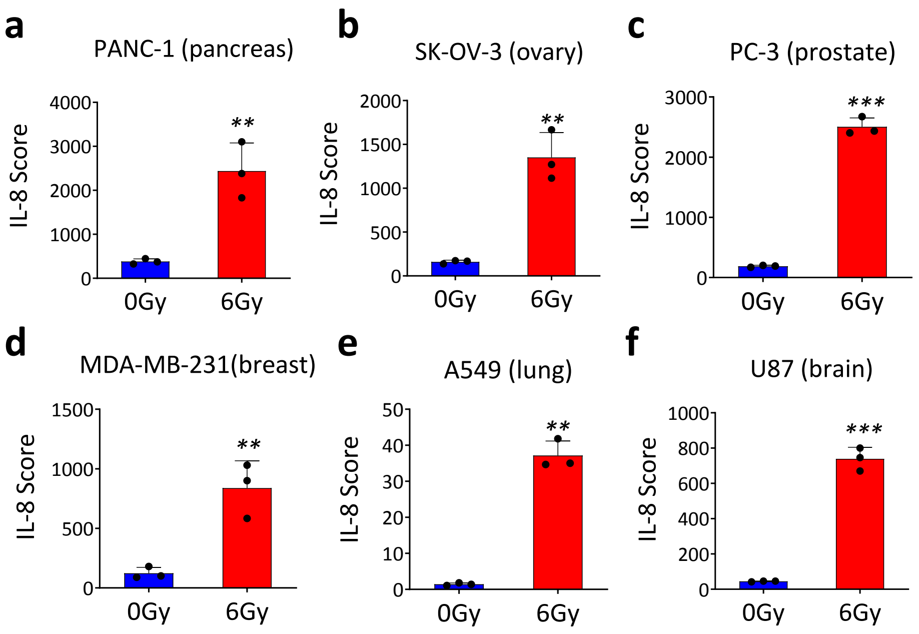

Supplementary Fig. 1. Enhanced expression of IL-8 after exposure to ionizing radiation across multiple human cancer types. a-e. IL-8 protein expression of multiple tumor cell lines 7 days after treatment with 6 Gy irradiation. Tumor cells were radiated at the indicated dose and seeded at $1x10^4$/ml. The supernatant was harvested 7 days after radiation, and the IL-8 secretions (means ± SD; n = 3) were measured by ELISA. The number of cells was determined using counting beads, and propidium iodide (PI) was used to gate viable cells. The IL-8 score represents a normalization of concentration to viable cells. The Mann–Whitney U test was used for the difference between 2 groups.

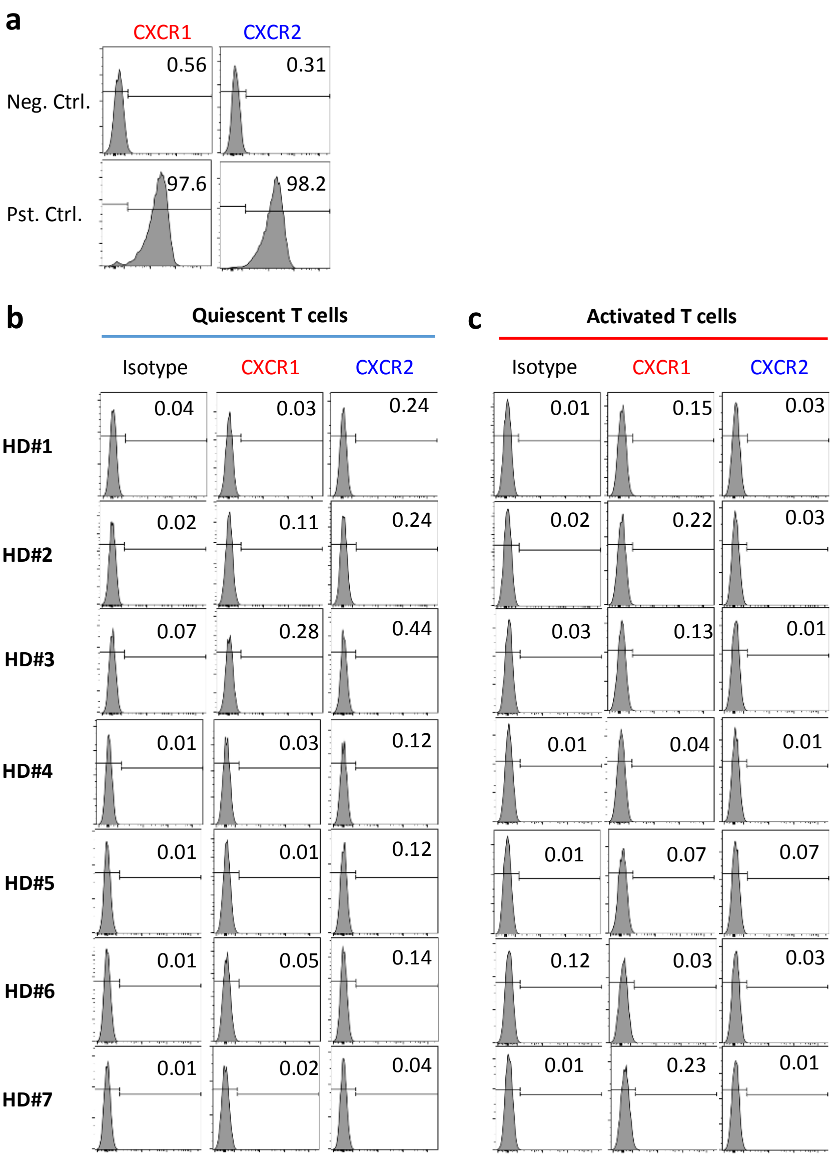

Supplementary Fig. 2. No IL-8R expression was detected on human T cells. a-c. Flow cytometric analysis of IL-8 receptor (CXCR1 and CXCR2) expressions on quiescent and activated T cells. PBMCs were isolated by Ficoll–Paque density gradient centrifugation from healthy donor buffy coat (n = 7) and activated with anti-CD3/CD28 Dynabeads (bead: cell = 3:1) in the presence of 100 IU/ml IL-2 for 10 days. CD3^{+} and PI negative cells were gated, and CXCR1 and CXCR2 were evaluated by flow cytometry analysis. The Jurkat cell line was used as the negative control, and FACS-sorted CAR-R1 and CAR-R2 T cells were used as the positive control, whereas isotype control was included for gating.

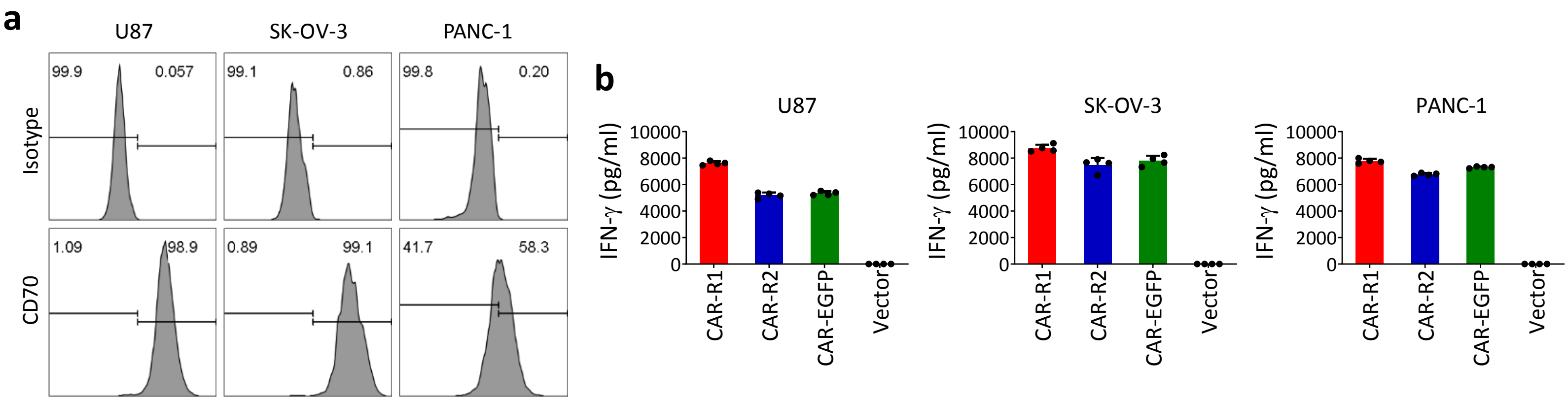

Supplementary Fig 3. Comparable tumor recognition of the 8R-mod-70CARs and un-mod-70CAR T cells. a. The expression level of CD70 on U87, SK-OV-3, and PANC-1 cell lines. Flow cytometry analysis was performed for the CD70 expression. The gates were drawn based on the isotype control. **b.** T cell recognition of the 8R-mod-70CARs and un-mod-70CAR T cells *in vitro*. The CAR T cells ($1x10^5$) were respectively cocultured with $1x10^5$ of U87, SK-OV-3, and PANC-1 tumor cells for overnight, and the supernatants were collected to assess IFN-γ production, measured by ELISA. The data represent mean ± SD and are representative of 3 independent experiments.

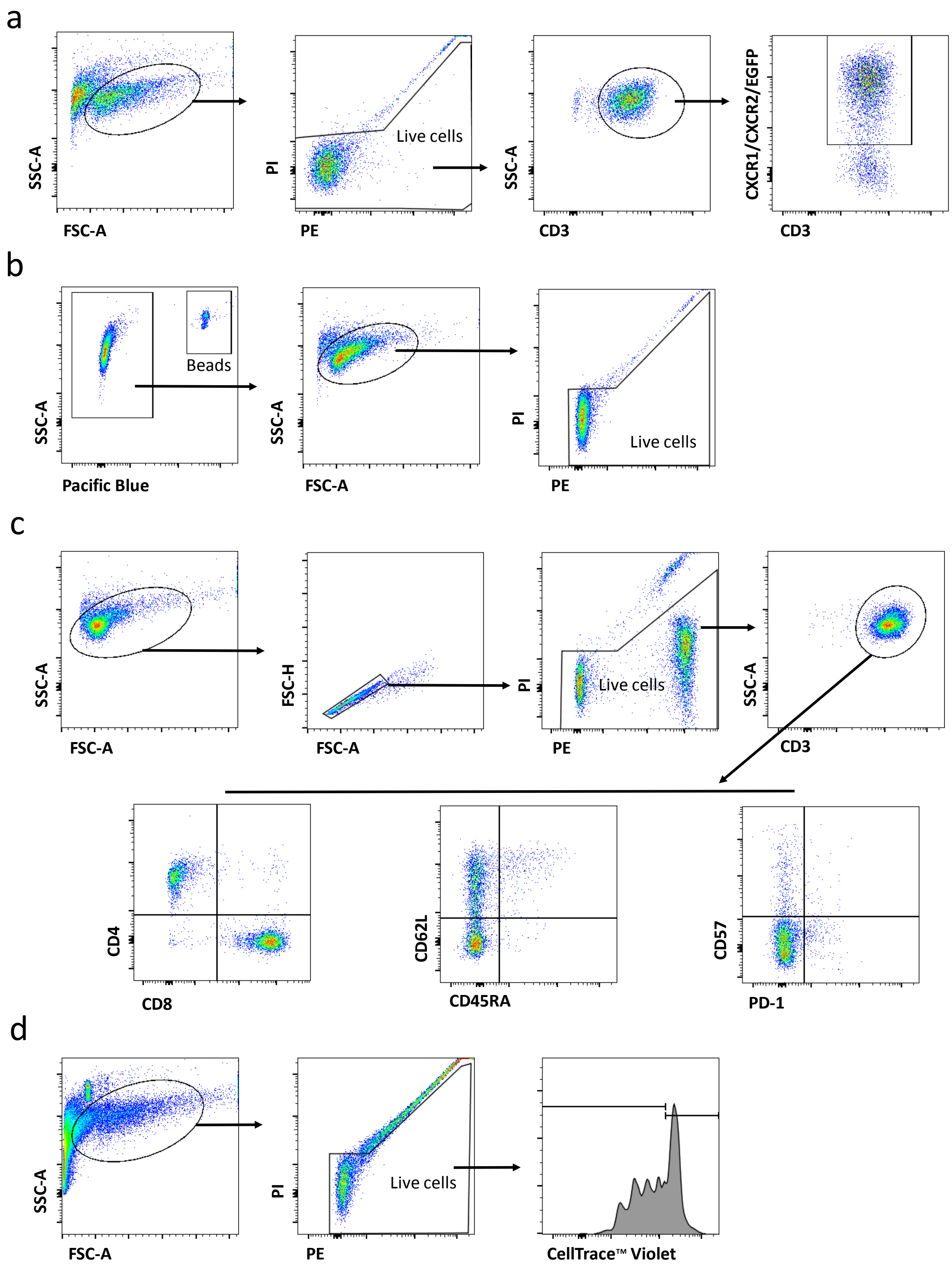

a
SSC-A
FSC-A
PI
PE
Live cells
SSC-A
CD3
CXCR1/CXCR2/EGFP
CD3
b
SSC-A
Pacific Blue
Beads
SSC-A
FSC-A
PI
PE
Live cells
c
SSC-A
FSC-A
FSC-H
FSC-A
PI
PE
Live cells
SSC-A
CD3
CD4
CD8
CD62L
CD45RA
CD57
PD-1
d
SSC-A
FSC-A
PI
PE
Live cells
CellTrace™ Violet

e

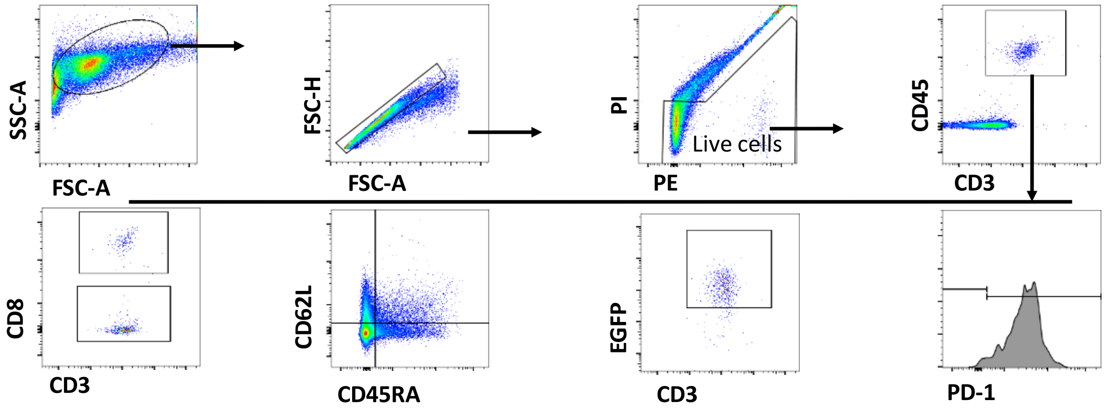

f

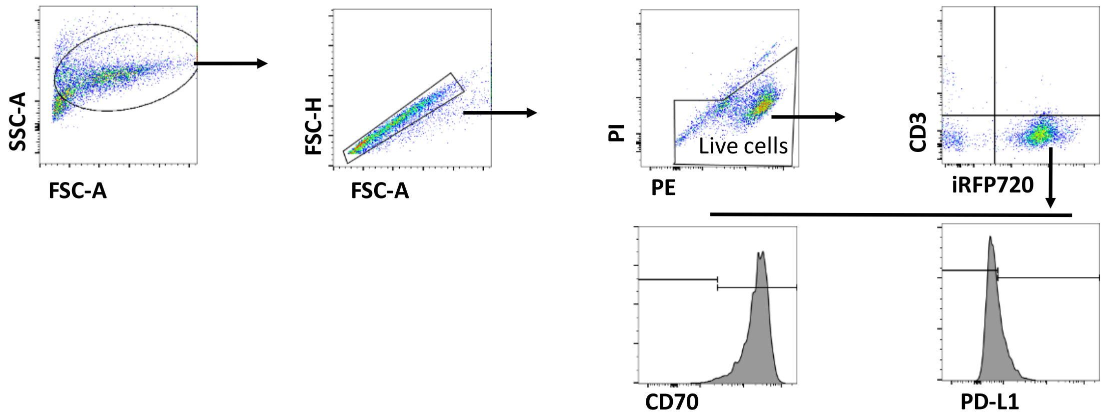

Supplementary Fig. 4. Flow cytometry gating strategies used in Fig.2 and Fig. 5. **a.** Gating strategies for analyzing T cell transduction efficiency of CAR-R1(CXCR1$^+$), CAR-R2 (CXCR2$^+$) and CAR-EGFP (EGFP$^+$) T cells in **Fig. 2a**. **b.** Cell counts in **Fig. 2c** were measured by the flow cytometer using CountBright™ absolute counting beads. Beads were distinguished by fluorescence signal (Pacific blue$^+$), and side scatter and live (PI$^-$) cells were counted. **c.** Gating strategies to analyze CD4+ T cell (CD4$^+$CD8$^-$), CD8+T cell (CD4$^-$CD8$^+$), naïve T cell (CD62L$^+$CD45RA$^+$), central memory T cell (CD62L$^+$CD45RA$^-$), effector memory T cell (CD62L$^-$CD45RA$^-$), effector T cell (CD62L$^-$CD45RA$^+$) and exhaustion markers (CD57+, PD-1+) in **Fig. 2d**. **d.** Cell proliferation was distinguished by fluorescent dye dilution on live (PI$^-$) cells in **Fig. 2i**. **e.** T cells (CD3+CD45+) from tumor, spleen or before adoptive transfer were gated to analyze CD4$^+$T cell (CD3$^+$CD8$^-$), CD8$^+$T cell (CD3$^+$CD8$^+$) in **Fig. 5d**, , naïve T cell (CD62L+CD45RA+), central memory T cell (CD62L+CD45RA-), effector memory T cell (CD62L-CD45RA-), effector T cell (CD62L-CD45RA+) in **Fig. 5e**, CAR-EGFP T cell percentage (EGFP$^+$) in **Fig. 5f**, and PD-1 expression on T cell in **Fig. 5g**. **f.** CD3$^-$iRFP720$^+$ tumor cells are gated to analyze tumor CD70 in **Fig.5i** and PD-L1 expression in **Fig5j**.

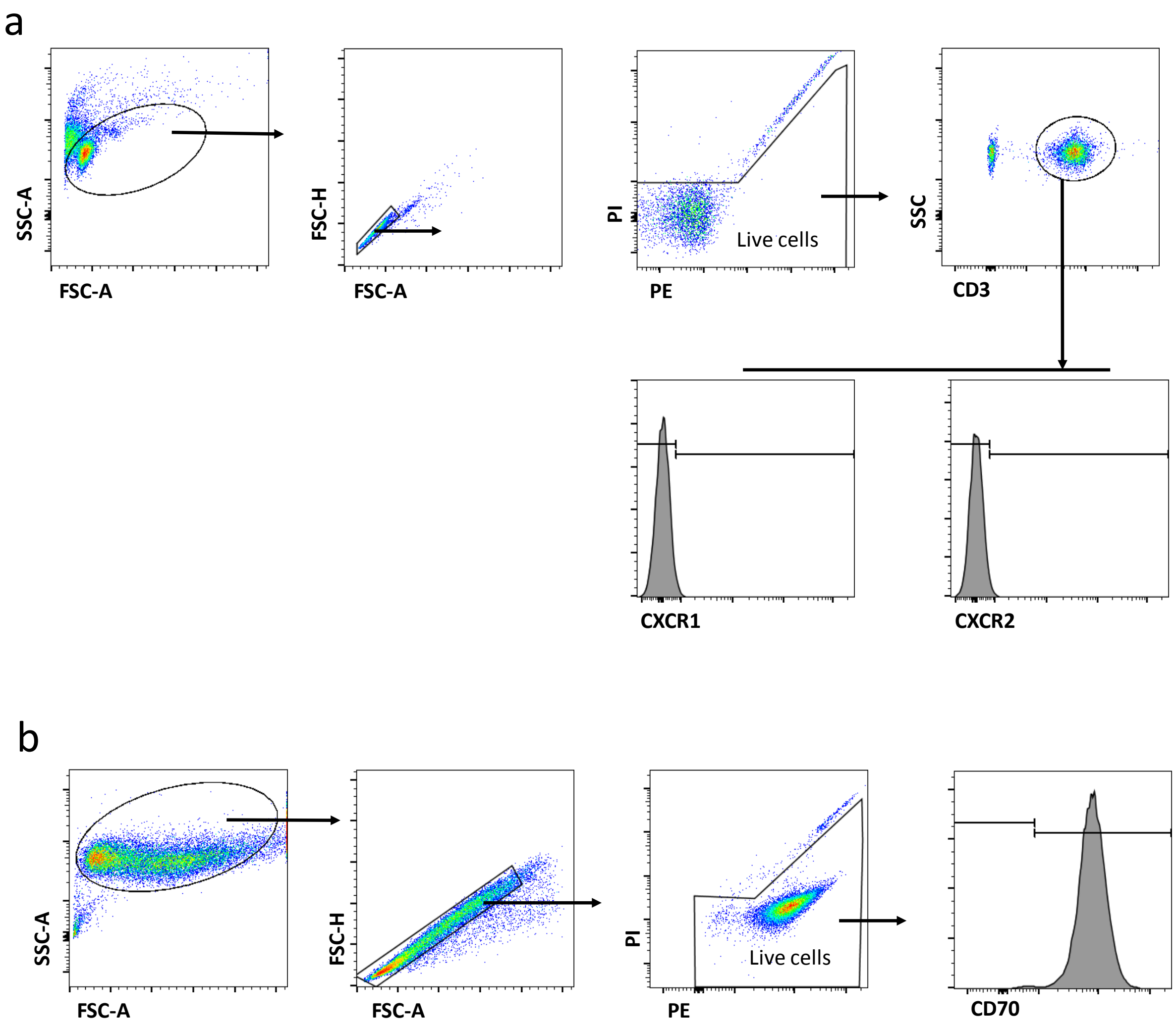

Supplementary Fig. 5. Flow cytometry gating strategies used in the Supplementary Figures. a. Gating strategies to analyze T cell CXCR1 or CXCR2 expression in **Supplementary Fig. 2**. **b.** Gating strategies to analyze tumor cell CD70 expression in **Supplementary Fig. 3**. The gate for CXCR1⁺, CXCR2⁺, or CD70⁺ cells was based on the isotype control.

Supplementary Table 1. Primers used in this study

Primer/Product	Catalog#	Assay method
Cytokines and chemokines (SAB Target List) H384,<br>Predesigned 384-well panel for use with SYBR® Green	Bio-Rad(10034472)	SYBR® Green
Human IL-8	ThermoFisher Scientific (Hs00174103_m1)	TaqMan®
Human RPL13A	ThermoFisher Scientific (Hs01578912_m1)	TaqMan®

Supplementary Table 2. Antibodies used in this study

Antibodies	Company	Catalog#	Application	Dilution
BV421 Mouse Anti-Human CD3	BD Biosciences	563798	Flow	1:50
APC Mouse Anti-Human CD181 (CXCR1)	BD Biosciences	551080	Flow	1:20
PE Mouse Anti-Human CD182 (CXCR2)	BD Biosciences	555933	Flow	1:50
APC-Cy™7 Mouse Anti-Human CD4	BD Biosciences	557871	Flow	1:50
PE Mouse Anti-Human CD8	BD Biosciences	555635	Flow	1:20
APC Mouse Anti-Human CD62L	BD Biosciences	561916	Flow	1:50
PE-Cy™7 Mouse Anti-Human CD45RA	BD Biosciences	561216	Flow	1:50
APC Mouse Anti-Human CD57	BD Biosciences	560845	Flow	1:50
PE-Cy™7 Mouse anti-Human CD279 (PD-1)	BD Biosciences	561272	Flow	1:50
APC-H7 Mouse Anti-Human CD3	BD Biosciences	560176	Flow	1:50
APC Mouse Anti-Human CD4	BD Biosciences	555349	Flow	1:20
BV421 Mouse Anti-Human CD45	BD Biosciences	563879	Flow	1:50
PE Mouse Anti-Human CD70	BD Biosciences	555835	Flow	1:20
APC Mouse Anti-Human CD274 (PD-L1)	BD Biosciences	563741	Flow	1:50

FITC Mouse Anti-Human CD181 (CXCR1)	BD Biosciences	555939	Flow	1:50
FITC Mouse Anti-Human CD182 (CXCR2)	BD Biosciences	551126	Flow	1:20
Mouse Anti-Human IL-8	BD Biosciences	554726	Neutralization	1:250
Rabbit anti-human IL-8	Abcam	ab7747	IHC	1:50
Goat anti-rabbit HRP polymer	Vector Laboratories Inc.	MP-7451	IHC	Ready-to-use
Rat anti-human CD45	Thermo Fisher Scientific	MA5-17687	IF	1:500
Rabbit anti-human Granzyme B	Abcam	ab134933	IF	1:100
Rabbit anti-human PD1	Novus Biologicals	NBP2-47621-0.5ML	IF	1:100
Goat anti-rat IgG-Alexa Fluor488	Thermo Fisher Scientific	A-11006	IF	1:500
Goat anti-rabbit IgG-HRP	ThermoFisher Scientific	B40922	IF	Ready-to-use
